# *NGN2* mmRNA-Based Transcriptional Programming in Microfluidic Guides hiPSCs Toward Neural Fate With Multiple Identities

**DOI:** 10.3389/fncel.2021.602888

**Published:** 2021-02-12

**Authors:** Anna Maria Tolomeo, Cecilia Laterza, Eleonora Grespan, Federica Michielin, Isaac Canals, Zaal Kokaia, Maurizio Muraca, Onelia Gagliano, Nicola Elvassore

**Affiliations:** ^1^Department of Industrial Engineering, University of Padua, Padua, Italy; ^2^L.i.f.e.L.a.b. Program, Consorzio per la Ricerca Sanitaria (CORIS), Padua, Italy; ^3^Veneto Institute of Molecular Medicine, Padua, Italy; ^4^Institute of Neuroscience, National Research Council, Padua, Italy; ^5^Stem Cells, Aging and Neurodegeneration Group, Lund Stem Cell Center, Lund University, Lund, Sweden; ^6^Laboratory of Stem Cells and Restorative Neurology, Lund Stem Cell Center, Lund University, Lund, Sweden; ^7^Department of Women’s and Children’s Health, Faculty of Medicine, University of Padua, Padua, Italy

**Keywords:** *NGN2*, induced neurons, microfluidics, mmRNA, transcriptional programming

## Abstract

Recent advancements in cell engineering have succeeded in manipulating cell identity with the targeted overexpression of specific cell fate determining transcription factors in a process named transcriptional programming. Neurogenin2 (NGN2) is sufficient to instruct pluripotent stem cells (PSCs) to acquire a neuronal identity when delivered with an integrating system, which arises some safety concerns for clinical applications. A non-integrating system based on modified messenger RNA (mmRNA) delivery method, represents a valuable alternative to lentiviral-based approaches. The ability of NGN2 mmRNA to instruct PSC fate change has not been thoroughly investigated yet. Here we aimed at understanding whether the use of an NGN2 mmRNA-based approach combined with a miniaturized system, which allows a higher transfection efficiency in a cost-effective system, is able to drive human induced PSCs (hiPSCs) toward the neuronal lineage. We show that NGN2 mRNA alone is able to induce cell fate conversion. Surprisingly, the outcome cell population accounts for multiple phenotypes along the neural development trajectory. We found that this mixed population is mainly constituted by neural stem cells (45% ± 18 PAX6 positive cells) and neurons (38% ± 8 βIIITUBULIN positive cells) only when NGN2 is delivered as mmRNA. On the other hand, when the delivery system is lentiviral-based, both providing a constant expression of NGN2 or only a transient pulse, the outcome differentiated population is formed by a clear majority of neurons (88% ± 1 βIIITUBULIN positive cells). Altogether, our data confirm the ability of NGN2 to induce neuralization in hiPSCs and opens a new point of view in respect to the delivery system method when it comes to transcriptional programming applications.

## Introduction

The derivation of patient-specific human induced pluripotent stem cells (hiPSCs) has opened new prospective in terms of disease modeling and treatment options for many orphan diseases. Neurological disease represents one of the best candidates to be modeled *in vitro* by hiPSC technology, since human brain tissue is difficult to obtain, and can benefit from the derivation of neural stem cells (NSCs) from hiPSCs for cell replacement strategies (Bahmad et al., 2017; Mertens et al., 2018).

There are two main strategies to obtain NSCs and neurons from hiPSCs. The first goes step-by-step through a series of stages that recapitulate human brain development cues. In this approach hiPSC differentiation involves the generation of neuroectoderm and NSC formation via inhibition of the bone morphogenetic protein (BMP) and Activin/TGFβ signaling pathways (Chambers et al., 2009; Maroof et al., 2013). Then, NSCs are terminally differentiated by a combination of patterning molecules (i.e., small molecules and growth factors). However, these procedures are limited in speed and scale and are typically complex protocols that involve multiple steps.

The second one involves a rapid and efficient differentiation by overexpression of specific transcription factors (TFs), which are master regulators of the cell lineage of interest (Son et al., 2011; Mertens et al., 2016). This last method, named TF programming, allows a faster generation of the target cell population bypassing or shortening many developmental stages that cell experiences during differentiation *in vivo* (Flitsch et al., 2020). The milestone in the TF programming was reached from Zhang and colleagues who proved that Neurogenin2 (NGN2) alone was able to program pluripotent stem cells into functional neuronal like-cells in 2 weeks (Zhang et al., 2013). Then, TF programming has been used to efficiently derive various types of functional neurons from PSCs, thanks to their ability of fine-tuning the specification of distinct neural subtypes (e.g., excitatory neurons (Zhang et al., 2013), inhibitory neurons (Yang et al., 2017), dopaminergic neurons (Theka et al., 2013), motor neurons (Goto et al., 2017).

To assure a high and continuous expression of the exogenous TFs, integrating systems are the most used approaches. However, the integration of foreign DNA in the host genome can lead to potential problems linked to genome modifications, random integrations in regulatory or coding sequences, difficulties in silencing the expression of exogenous transcript and uncontrolled expression level. Even if some of these hurdles have been addressed by countermeasures (such as TALENs or CRISPR/Cas9 strategies to better control integration sites), integration is strongly associated with safety limitations for any clinical translation (Flitsch et al., 2020).

To obtain a fast and efficient differentiation strategy without safety limitations, expression of exogenous TFs through delivery of modified messenger RNA (mmRNAs) represents a valuable alternative to the use of viral vectors (Goparaju et al., 2017). To some extent, the level of expression can be controlled by tuning the mmRNA concentration and, due to its short lifetime, exogenous expression can be stopped in 24 h (Warren et al., 2010), and activity of the endogenous master regulator of specific phenotypes can be properly evaluated (Xue et al., 2019). Thus, GMP-grade mmRNA programming can be properly achieved by controlling timing and dose.

However, the competitive advantages of the mmRNA-based method hold some intrinsic differences compared to integrating systems that can highly affect the expression of TF programming and, likely, the phenotypic outcomes: (i) mmRNA delivery generally requires daily transfections to maintain a balance between rapid translation and subsequent degradation, which are likely to result in daily fluctuations in the protein level contained in the transfected cells; (ii) the poor efficiency of mmRNA delivery based on cell transfection methods does not assure the maintenance of a high amount of translated protein as obtained with viral based methods (Xue et al., 2019).

Both fluctuations and low basal levels of TF expression, combined with a stochastic distribution of the mRNA in the target cells, could affect the overall efficiency of hiPSC programming resulting in multiple phenotypes along neural development trajectories.

In line with these observations, the only available papers using mRNA delivery for neuronal programming showed that it is possible to guide neuronal differentiation of PSCs toward a specific neuronal cell type only using a complex combination of multiple mRNAs coding for different TFs and small molecules and/or neuralization factors known to induce themselves neural commitment in PSCs (Goparaju et al., 2017; Xue et al., 2019).

It is well proven that NGN2 alone is sufficient to instruct PSCs to acquire a neuronal identity when delivered with integrating systems (Zhang et al., 2013), however, once NGN2 is delivered as mmRNA it seems that it needs the support of other TF and/or small molecules to assure an efficient transcriptional programming (Goparaju et al., 2017; Xue et al., 2019).

To our knowledge there are no reports showing neuronal programming using *NGN2* mmRNA only.

Here, we investigated whether the use of mmRNA-based delivery method for NGN2 TF can support neuronal induction and if the intrinsic fluctuating nature of mmRNA, originating from the need of daily transfections, leads to the generation of multiple neural phenotypes.

We designed a single TF based neural programming using *NGN2* mmRNA. To assure an efficient delivery of mmRNA, we took advantage of a microfluidic environment that we developed in our lab and we previously showed to significantly enhance transfection efficiency in a reprogramming setting (Luni et al., 2016; Gagliano et al., 2019).

We optimized the protocol by perturbating both FGF2 and NOTCH signaling pathways and we obtained the generation of both NSCs (PAX6, NESTIN, and SOX2 positive) and neurons (βIIITUBULIN, NEUN, and MAP2 positive). We found that the generation of a distinct NSC population is a specific feature of mmRNA based NGN2 transcriptional programming not shared with lentiviral based methods. We also confirmed that a single pulse of NGN2 TF delivered with lentiviral system was not able to mimic mmRNA delivery system. These data suggest that the intrinsic fluctuations in mmRNA and protein levels generated via mmRNA transfection are able to establish an oscillatory-like NGN2 pattern, which results in the formation of a NSC population.

## Results

### Set Up of mmRNA Based Neuronal Programming in Microfluidics

**FIGURE 1:**
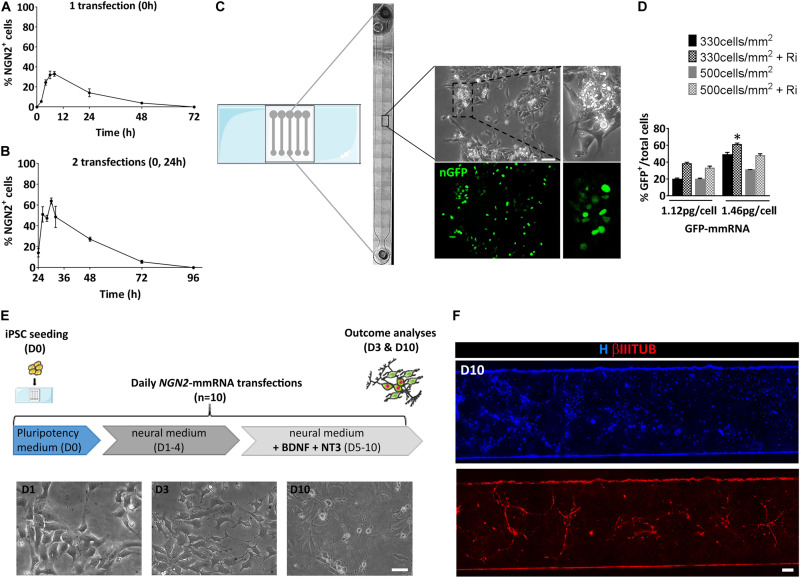
Setting of the experimental conditions for NGN2 mRNA based transcriptional programming of hiPSCs. **(A)** Time course of NGN2 protein expression evaluated by means of immunofluorescence after a single *NGN2* mmRNA transfection in hiPSCs. **(B)** Time course of NGN2 protein production evaluated by means of immunofluorescence two *NGN2* mmRNA transfections in hiPSCs. **(C)** Representative image of the microfluidic platform used in our experiments. Shown is the reconstruction of a single microfluidic channel in bright field with the magnification of an area of hiPCSs transfected with GFP mRNA (green). **(D)** Percentage of GFP-positive hiPSCs after transfection with GFP mmRNA combining different cell densities, mmRNA doses and the presence of Rock-inhibitor (Ri). *n* = 3 independent replicates (mean ± SEM). *P*-values: *p < 0.05. **(E)** Top: Experimental outline. hiPSCs were seeded at Day 0 (D0) in pluripotency medium in the microfluidic chambers and transfected daily with NGN2 mmRNA. Medium was replaced with neural medium from Day 1 until Day 3 (D1–3) and supplemented with neurotrophic factors from Day 5 till Day 10 (D5–10). The outcome analysis was performed at Day 3 and Day 10 (D3 and D10). Bottom: Representative bright field images of hiPSCs after 1, 3, and 10 days of transfection with NGN2 mmRNA (scale bar = 100 μm). **(F)** Representative image of a single microfluidic channel of hiPSCs at Day 10 of NGN2 mmRNA transcriptional programming. Shown are nuclei (blue) and neurons (red). The image was created merging single microscopy images next to each other (scale bar = 1 mm); ns, not significant.

Before evaluating the ability of *NGN2*-mmRNA to induce neuronal programming, we examined the kinetic of NGN2 expression after a single transfection with *NGN2* mmRNA by means of immunofluorescence. Production of NGN2 protein was visible as early as 2 h after transfection with *NGN2*-mmRNA and a peak of NGN2 positive cells was observed between 8 and 24 h after transfection (Figure 1A). After reaching the peak, the percentage of NGN2 positive cells gradually decreased and reached half of the number around 24 h after transfection, disappearing at 72 h after transfection (Figure 1A). Given the transient nature of proteins generated by mmRNA transfection and the rapidity of exogenous mmRNA clearance (Sul et al., 2012), to assure a sustained NGN2 expression we transfected cells once per day, as reported for reprogramming procedure (Luni et al., 2016; Gagliano et al., 2019). We further investigated the effect of repeated daily transfections on the percentage of NGN2 positive cells. We found that after the second transfection (Figure 1B) the percentage of NGN2 positive cells increased with a similar behavior observed after a single transfection (Figure 1A), but reaching almost the double of NGN2 positive cells than the one obtained after a single transfection (Figure 1B). At 72 h after the second transfection (96 h in Figure 1B), the percentage of NGN2 positive cells dropped to the basal level (Figure 1B) showing the fast decay of mmRNA and supporting the need of multiple daily transfections.

We took advantage of a miniaturized system known to improve mmRNA delivery and to promote a faster accumulation of cellular extrinsic endogenous factors that further enhances the reprogramming efficiency (Giulitti et al., 2013; Luni et al., 2016; Gagliano et al., 2019; Figure 1C). We tested the optimal cell density and mmRNA concentration that ensures the highest number of transfected cells with a single daily transfection inside the microfluidic device using mmRNA encoding a nuclear-targeted green fluorescence protein for a total of 7 days (nGFP) (Figures 1C,D). We tested two cell densities (330 and 500 cells/mm^2^) and two mmRNA concentrations (1.12 and 1.46 pg/cell) with or without rock-inhibitor (Ri, Y27632) treatment that, acting on cell cytoskeleton, increases the cell surface available for mmRNA delivery resulting in improved cell transfection (Yen et al., 2014; Figure 1D). Among all the different combinations tested, the condition where we seeded 330 cell/mm^2^ and transfected with 1.46 pg/cell of mmRNA, giving 60.9% ± 2.9 GFP positive cells after 7 days of transfection, resulted the more efficient in terms of number of transfected cells and was the one selected for all the subsequent experiments (Figure 1D).

Based on these preliminary considerations, we designed the NGN2-mediated transcriptional programming experiment in microscale as shown in Figure 1E. Briefly, a single-cell suspension of hiPSCs was seeded in the microfluidic chamber in a medium that sustains pluripotency (Essential 8, E8) and in the presence of the transfection reagent and *NGN2*-mmRNA. After 8 h, medium was changed to fresh E8 with Ri and B18R protein, to reduce interferon response. From Day 1 to Day 4, transfections were performed in a neuronal basal medium (N2/B27 medium). The last 5 days, N2/B27 medium was supplemented with growth factors known to promote neuronal survival and maturation (e.g., NT3 and BDNF). During time, hiPSCs transfected with *NGN2*-mmRNA underwent an epithelial to mesenchymal transition and part of them acquired a neuronal-like morphology (Figures 1E,F).

### *NGN2*-mmRNA Induces Generation of Both Neurons and NSCs in hiPSCs

**FIGURE 2:**
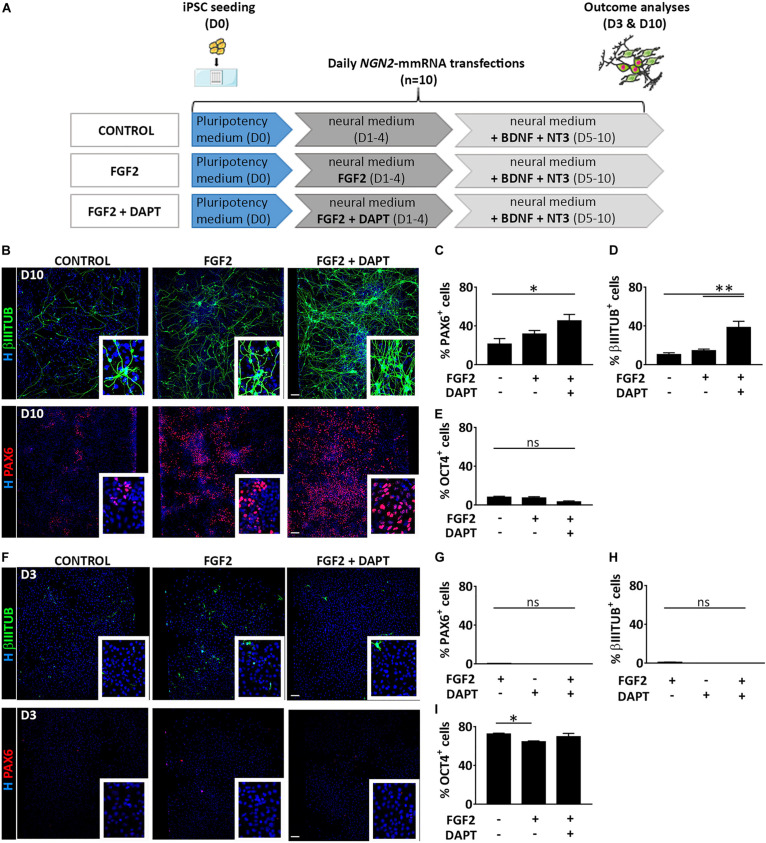
Treatment with FGF2 and DAPT improves neural conversion efficiency. **(A)** Experimental design. Note that FGF2 was used only from Day 1 to Day 4 and DAPT from Day 1 till the end of the differentiation protocol (Day 10). **(B)** Representative images of βIIITUBULIN (top) and PAX6 (bottom) staining at Day 10 in the presence or absence of FGF2 and DAPT. Nuclei are counterstained with Hoechst (blue) (scale bar = 100 μm). **(C–E)** Percentage of positive cells for PAX6 **(C)**, βIIITUBULIN **(D)** and OCT4 **(E)** markers over the total cells at Day 10 in all the conditions. *n* = 3 independent replicates (mean ± SEM) **(F)** Representative images of βIIITUBULIN (top) and PAX6 staining at Day 3 in the presence or absence of FGF2 and DAPT (scale bar = 100 μm). **(G–I)** Percentage of positive cells for PAX6 **(G)**, βIIITUBULIN **(H)** and OCT4 **(I)**, markers at Day 10 in all the conditions. *n* = 3 independent replicates (mean ± SEM). *P*-values: *p < 0.05; **p < 0.01; ns, not significant.

We thoroughly investigated the outcome cell population derived from *NGN2*-mmRNA based transcriptional programming of hiPSCs. After 10 days of *NGN2*-mmRNA transfection (Figure 2A) we observed that, in the absence of any growth factor/inhibitor, we obtained a mixed population of NSCs (27.1% ± 13.3 PAX6 positive cells) and neurons (10.4% ± 4.3 βIIITUBULIN positive cells) (Figures 2B–D), while still having few pluripotent cell (7.9% ± 2.6 OCT4 positive cells) (Figure 2E and Supplementary Figure 1) meaning that NGN2 alone, delivered as mmRNA, was able to promote neurogenesis in hiPSCs.

We investigated if the addition of FGF2 during the first days of our neuronal induction protocol (days 1–3) was able to increase the generation of neural-like cells (Figure 2A). FGF2 is a growth factor with pleiotropic effects, crucial both in human PSC maintenance, central nervous system development and adult neurogenesis (Woodbury and Ikezu, 2014; Mossahebi-Mohammadi et al., 2020). We found that in this condition, at Day 10, both the NSC population (31.5% ± 10.0 PAX6 positive cells) and the neuronal population (14.6% ± 3.6 βIIITUBULIN positive cells) slightly increased (Figures 2B–D) while few pluripotent cells were still present (7.1% ± 3.4 OCT4 positive cells) (Figure 2E and Supplementary Figure 1). To further improve the *NGN2* mmRNA-mediated neurogenesis we decided to act on Notch signaling pathway using DAPT, an inhibitor of the Notch-activating enzyme γ-secretase. Notch signaling plays an important role in the maintenance of NSCs and regulates neuronal differentiation (Shimojo et al., 2011). Notch effector, HES1, promotes maintenance of NSCs and repress NGN2 expression, inhibiting neuronal differentiation. Blocking Notch signaling via DAPT treatment resulted in HES1 blockage, further promoting NGN2 endogenous activity. Using this approach, we were able to significantly increase the conversion efficiency of hiPSCs into PAX6 (45.1% ± 18.7) and βIIITUBULIN (38.3% ± 8.6) positive cells (Figures 2B–D).

At an earlier time point (Day 3 of transfection) no neurons nor NSCs were visible in none of the three conditions, but cells were mostly pluripotent (72.2% ± 2.4 no factors, 64.5% ± 2.1 + FGF2, 69.5% ± 8.0 + FGF2 + DAPT OCT4 positive cells) (Figures 2F–I and Supplementary Figure 1).

On the other side, at a later time point (10 days after the last transfection, total of 20 days in culture, D20) (Supplementary Figure 2A), the microenvironment in the microfluidic system was able to support NSCs proliferation (63.8% ± 23.4 no factors, 55.5% ± 18.4 + FGF2, 61.9% ± 26.8 + FGF2 + DAPT PAX6 positive cells) (Supplementary Figures 2B,C). Due to their high proliferation rate, the NSCs took over the post-mitotic neuronal population (0.7% ± 0.7 no factors, 1.7% ± 1.7 + FGF2, 10.3% ± 11.2 + FGF2 + DAPT βIIITUBULIN positive cells) (Supplementary Figures 2B–D). Surprisingly, a small fraction of OCT4 positive cells is still present (9.2% ± 13.6 no factors, 45.5% ± 32.9 + FGF2, 26.1% ± 25.9 + FGF2 + DAPT OCT4 positive cells) (Supplementary Figures 1, 2B,E).

As control, we evaluated the presence of neural cells in the microfluidic culture of hiPSCs subjected to the same culture conditions described above but without mmRNA transfections (Supplementary Figure 3A). No PAX6 or βIIITUBULIN positive cells were detected either at Day 3 or at Day 10 culturing hiPSCs in neuronal medium with FGF2 and DAPT in the absence of *NGN2*-mmRNA transfection (Supplementary Figure 3B) indicating that the medium and the growth factors/inhibitor themselves are not able to induce neural commitment of hiPSCs. This suggests that, to improve neural induction mediated by *NGN2*-mmRNA, both FGF2 and Notch inhibitor play an important role but only if combined to NGN2 delivery. Therefore, in all further experiments we supplemented the media with this combination of molecules.

**FIGURE 3:**
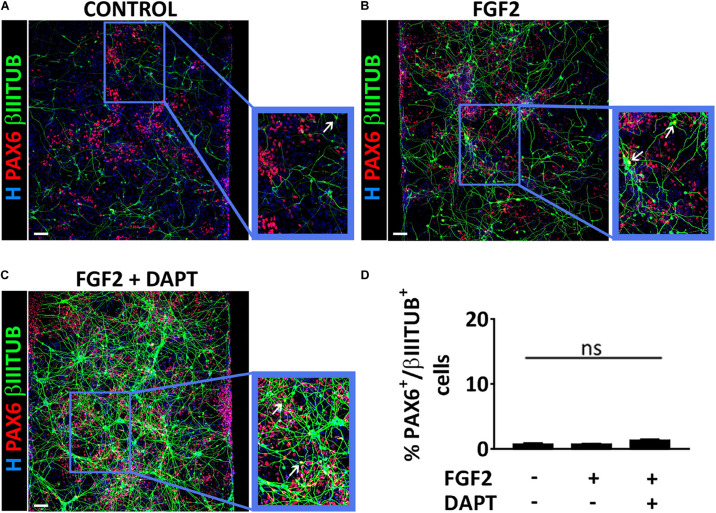
PAX6 and βIIITUBULIN positive cells represent two distinct cell populations. **(A–C)** Representative images of a co-stain between PAX6 (red) and βIIITUBULIN (green) at Day 10 of differentiation in the control condition **(A)**, in the presence of FGF2 **(B)** and in the presence of both FGF2 and DAPT **(C)** (scale bar = 100 μm). White arrows indicate cells double positive for PAX6 and βIIITUBULIN. **(D)** Percentage of cells double positive for PAX6 and βIIITUBULIN markers over the total number of βIIITUBULIN positive cells analyzed at Day 10. *n* = 3 independent replicates (mean ± SEM); ns, not significant.

To better characterize the cell populations obtained in our microfluidic device via mmRNA transfections, in all the conditions mentioned above we analyzed the co-localization of PAX6 and βIIITUBULIN markers to address whether these two markers were identifying two distinct cell types (i.e., NSCs and neurons) or were labeling a single cell population of immature neurons. We found that the percentage of cells co-expressing the two markers was extremely low in all conditions (0.5% ± 1.2 control, 1.0% ± 1.5 FGF2, 1.2% ± 0.9 FGF2 and DAPT) (Figures 3A–D), confirming the presence of two distinct cell populations, namely NSCs, and neurons.

**FIGURE 4:**
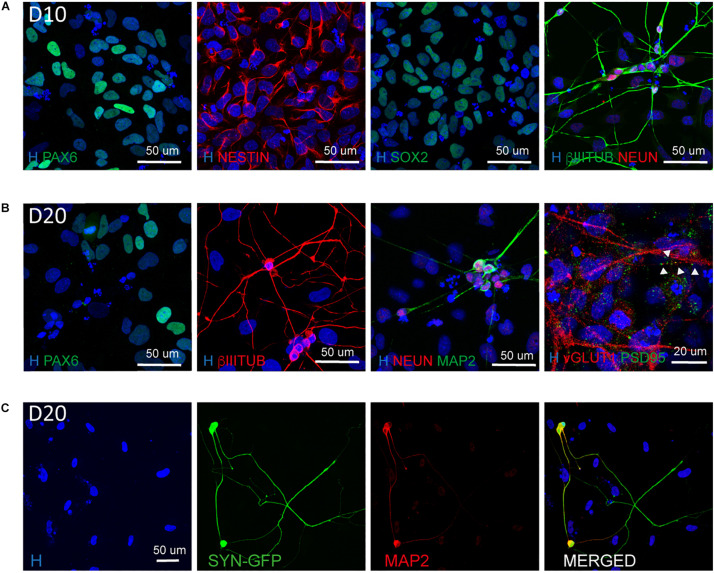
Characterization of the NSC and neuronal population at D10 and D20. **(A)** Representative images of NSC (PAX6, NESTIN, SOX2) and neuronal (βIIITUBULIN and NEUN) markers at Day 10 of differentiation (scale bar = 50 μm). **(B)** Representative images of NSC (PAX6), neuronal (βIIITUBULIN, MAP2, and NEUN) and synaptic (vGLUT1 and PSD95) markers at Day 20 of differentiation. The presence of synapses is highlighted by arrow heads (scale bar = 50 or 20 μm). **(C)** Representative images of neurons derived from Synapsin I-GFP iPSC line transfected 10 days with NGN2 mmRNA and kept in culture for additional 10 days (D20). Shown are MAP2 positive neurons expressing SYNI-GFP (scale bar = 50 μm).

In addition, we confirmed the NSC nature of the cells present in culture by their positivity for NESTIN, SOX2, and PAX6 (Figure 4A) and the presence of mature neurons both at D10 and D20 (Figures 4A–C). In particular at D20 we observed the expression of mature neuronal markers such as MAP2 and NEUN as well as the synaptic markers vGLUT1 and PSD95 indicating the presence of synapses (Figure 4B).

To understand the dynamic of neuronal maturation and synapses formation, we infected our hiPSC line with a lentiviral vector expressing GFP under the control of Synapsin I promoter and transfected this line with *NGN2*-mmRNA. We followed the expression of Synapsin I via the GFP reporter and we started observing GFP expression around 18–20 days in culture, suggesting that the neurons generated started expressing Synapsin I (Figure 4C). We confirmed the neuronal identity of these cells via MAP2 staining (Figure 4C).

Collectively, these results suggest that, mmRNA mediated neural programming is able to induce the generation of both mature neurons and NSCs.

### The Generation of NSCs Is a Specific Feature of NGN2 mmRNA-Based Transcriptional Programming

**FIGURE 5:**
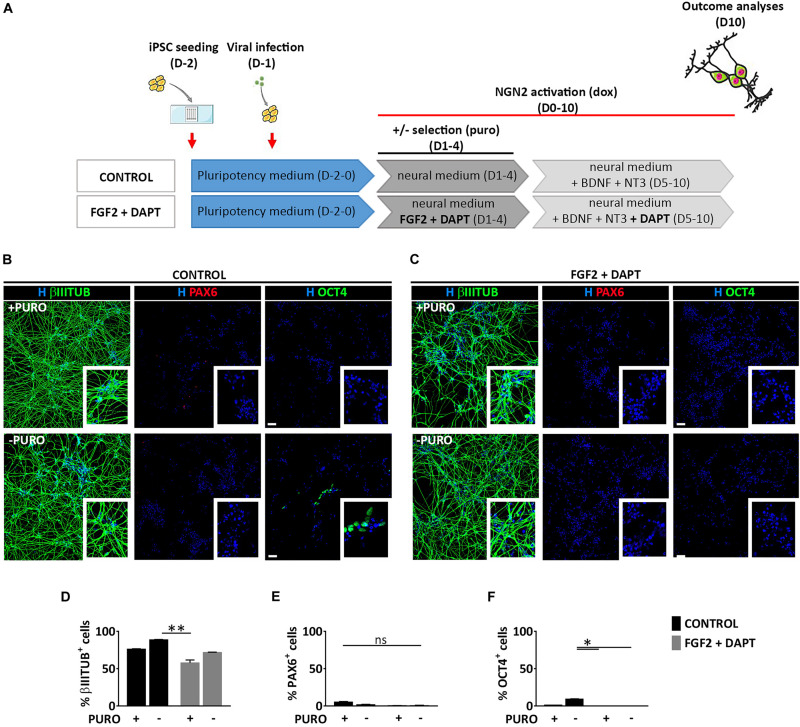
Lentiviral mediated transcriptional programming does not lead to the generation of PAX6 positive cells. **(A)** Experimental design. hiPSCs are seeded at Day-2 (D-2) in the microfluidic chamber and infected with dox inducible lentiviral vector coding for Ngn2. From Day 0 to Day 10 Ngn2 expression was activated by dox treatment. Infected cells were selected with puro treatment from Day 1 to Day 4. **(B,C)** Representative images of induced neural cells at Day 10, treated (top) or not (bottom) with puro, in the absence (**B**, CONTROL) or presence (**C**, FGF2 + DAPT) of FGF2 and DAPT. Shown are staining for βIIITUBULIN (red), PAX6 (red) and OCT4 (green) markers counterstained with Hoechst (H, blue) (scale bar = 200 μm) **(D–F)** Percentage of cells expressing βIIITUBULIN **(D)**, PAX6 **(E)**, and OCT4 **(F)** in all the conditions at Day 10. *n* = 3 independent replicates (mean ± SEM). *P*-values: *p < 0.05; **p < 0.01; ns, not significant.

To assess whether the generation of both NSCs (PAX6^+^) and neurons (βIIITUBULIN^+^), was a specific feature of mmRNA-based neural induction in the microfluidic device or was related to the combination of media and growth factors/inhibitors used to enhance neuronal conversion, we induced NGN2 overexpression in hiPSCs using a doxycycline (dox) inducible lentiviral construct (TetO-Ngn2-T2A-Puro) in our microfluidic platform. hiPSCs were plated at Day -2 in the microfluidic chambers at the same cell density used for mmRNA based experiments and infected 24 h later with a lentivirus carrying the constitutive expression of rtTA together with the TetO-Ngn2-T2A-Puro lentiviral vector. The following day (Day 0) Ngn2 expression was induced by exposure to dox, which was kept in the culture medium throughout the experiment. Puromycin (puro) was applied at Days 1–4 to select for transduced cells (Figure 5A).

Neuronal conversion was analyzed 10 days after Ngn2 overexpression, showing a very high efficiency with the number of βIIITUBULIN positive cells being almost 80% both with (74.9% ± 0.4) and without (88.6% ± 0.7) puromycin selection (Figures 5B,D), thus suggesting a very efficient lentiviral infection and neuronal conversion. This high number of neuronal cells was paralleled by a drastic reduction of OCT4 positive cells both with (1.0% ± 0.5) and without (9.3% ± 1.6) puromycin treatment and a very low percentage NSCs positive for PAX6 marker (5.3% ± 2.3 and 1.5% ± 1.3 with and without puro treatment, respectively) (Figures 5B,E,F), suggesting that the lentiviral-mediated overexpression of Ngn2 transcription factor induces a massive differentiation into neurons and not into NSCs.

To investigate whether FGF2 and DAPT had a central role on the appearance of a consistent PAX6 positive cell population observed in the mmRNA based experiments, we combined the lentiviral-based neuronal programming with DAPT-mediated Notch inhibition and FGF2 treatment keeping the same media composition and medium change rate (Figures 5A,C). We found that 10 days after Ngn2 induction, pluripotent cells were not detectable any more both with and without puro treatment (0% OCT4^+^) (Figures 5C,F) and βIIITUBULIN positive cells reached around 60% of the total cells (57.6% ± 8.1 and 71.8% ± 1.5 with and without puro treatment, respectively) (Figures 5C,D), which were significantly higher compared to mmRNA derived ones (Supplementary Figure 4A). However, this reduction in neuronal cells was not paralleled by an increase in NSCs, since the number of PAX6 positive cells was barely detectable (0.4% ± 0.2 and 0.6% ± 0.5 with and without puro treatment, respectively) (Figures 5C,E). In addition, when comparing the data obtained from the mmRNA based approach and the lentiviral based approach we found a statistical difference in terms of percentage of neurons and NSCs with NSCs present almost only in the mmRNA derived cells (Supplementary Figure 4B).

Once we confirmed that the generation of a NSC population was not related to the medium composition, we asked whether NGN2 expression dynamics could have an influence in the appearance of a PAX6 positive cell population.

Indeed, during development, sustained NGN2 expression promotes neuronal differentiation, while oscillatory expression maintains cells undifferentiated in a NSC state (Shimojo et al., 2011). Moreover, neuronal differentiation of one cell inhibits its neighboring cells from differentiating into the same cell type, thus maintaining a pool of stem/progenitor cells in a process named lateral inhibition (Shimojo et al., 2011).

**FIGURE 6:**
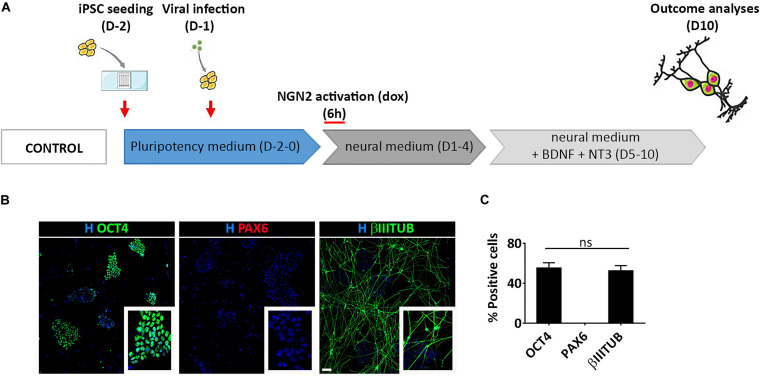
Transient activation of lentiviral mediated Ngn2 expression does not allow PAX6 positive cell appearance in culture. **(A)** Experimental design. Note that dox was provided only for 6 h in culture **(B)** Representative images of hiPSCs with a transient activation of Ngn2 stained for pluripotency (OCT4, green) neuronal (βIIITUBULIN, red) and NSC (PAX6, red) markers (scale bar = 100 μm). **(C)** Percentage of OCT4, PAX6, and βIIITUBULIN positive cells over the total number of cells at Day 10. *n* = 3 independent replicates (mean ± SEM); ns, not significant.

To gain a deeper understanding of this process we induced a transient activation of Ngn2 modulating dox administration in hiPSCs infected with TetO-Ngn2-T2A-Puro lentiviral vector (Figure 6A). In particular, we exposed hiPSCs to dox for 6 h and we analyzed the cells 10 days after induction. We found that a short-term induction of Ngn2 was able to promote hiPSC conversion into βIIITUBULIN positive cells (52.6% ± 7.1) (Figures 6B,C). However, even if only half of the cells become βIIITUBULIN positive, no PAX6 positive cells were detected and all the remaining cells were still pluripotent (55.0% ± 7.8 OCT4 positive cells) (Figures 6B,C).

Altogether, our results suggest that the viral mediated delivery of Ngn2 in hiPSCs leads to neuronal differentiation without creating a NSC population.

## Discussion

Here, we investigated whether the use of mmRNA as a delivery method for NGN2 TF was able to support neuronal transcriptional programming in hiPSCs.

In order to assure a high efficiency in mmRNA delivery for transcriptional programming, we further optimized the microfluidic platform, which was previously used for high-efficient transfection in mmRNA-based reprogramming (Giulitti et al., 2013; Luni et al., 2016; Gagliano et al., 2019). The use of microfluidics allows for a better delivery of mmRNA to target cells, including hiPSCs which have epithelial morphology and could be refractory to transfection. On the other hand, direct programming on a chip could have technological advantages because it provides an easily scalable system for disease modeling or drug screening applications (Zhang et al., 2020). In fact, the entire process from seeding hiPSC to neural induction is seamless, does not require intermediate cell sorting and low amount of media and mmRNA at microliter scale are needed, thus converting a quite expensive protocol in a cost-effective procedure.

We found that daily transfection of hiPSCs with *NGN2* mmRNA in a microfluidic system is sufficient to drive neuronal programming, even in the absence of any small molecules or patterning factors. Moreover, we found that acting both on FGF2 and Notch signaling pathways we obtained a significant increase in the neural conversion of hiPSCs.

This increase in the neuralization upon FGF2 treatment has been already reported in a different experimental setting, where they converted muller glia to retinal ganglion cells via NGN2 and ASCL1 overexpression (Guimarães et al., 2018). This can be explained, not only thanks to FGF2 role in NSC and PSC proliferation and differentiation (Woodbury and Ikezu, 2014), but also thanks to FGF2 ability to remodel chromatin and facilitate the binding of NGN2 to its target genes (Song and Ghosh, 2004).

Our results show that the combination of FGF2-treatment with DAPT further increases neural induction. DAPT is a gamma-secretase inhibitor that inhibits the activation of Notch signaling further enhancing neuronal differentiation (Borghese et al., 2010). Indeed, with the addition of DAPT we observed a significant increase in the number of neurons (βIIITUBULIN positive cells).

Surprisingly, we found that mmRNA-based delivery of NGN2 TF in hiPSCs is able to give rise to cells with multiple phenotypes along the neural development trajectory. In particular we obtained two distinct cell populations positive either for PAX6 or for βIIITUBULIN, representing a NSC population and a neuronal population, respectively.

The generation of such distinct populations can provide the possibility to generate, using a single transcription factor (i.e., NGN2), both neuronal cells and NSC that can be isolated and further expanded or instructed to acquire a terminal differentiated phenotype. In particular, NSCs can be guided to terminally differentiate not only into neurons but also into glia, which is known to be crucial for neuronal functionality, providing patient specific multicellular systems using a single transcription factor.

We then asked whether the generation of these two distinct populations was specific of mmRNA delivery method. In our experimental setting, we observed that the use of a lentiviral-based integrating system did not end up in generating such NSC population, but gave rise only to neuronal cells, in line with what has been previously reported. Indeed, it has been shown that forced expression of the NGN2 transcription factor induces rapid differentiation of hiPSCs into functional neuronal cells (Zhang et al., 2013).

One hypothesis for the appearance in culture of NSCs is that the direct differentiation passes through a stable NSC intermediate population, but this is still under debate since the expression of NSC markers results very short-lasting in lentiviral-based systems (Zhang et al., 2013; Busskamp et al., 2014). Another hypothesis is that the NSCs detectable in our experiment could result from an incomplete conversion during direct differentiation. In this respect, two reports showed a transient activation of NSC-like fate during neuronal conversion (Karow et al., 2018; Nehme et al., 2018). However, in the first case, authors started from a different cell type (pericytes) and used a different combination of transcription factors, including SOX2, a transcription factor also used to derive NSCs (Connor et al., 2018). In the second, authors combined lentiviral-based NGN2 programming with SMAD and WNT inhibition and obtained cells in different transcriptional states that ranged from early progenitor to well-differentiated excitatory neuron (Nehme et al., 2018).

In our work, it is clear that mmRNA-based delivery of NGN2 alone is able to generate a distinct population of PAX6 positive NSCs that we did not find using the lentiviral-based NGN2 overexpression. First, we excluded that the presence of this population of NSCs was caused by the microfluidic system, since when using the same experimental setting but combined with a lentiviral-delivery method, we did not observe the same phenomenon. These results indicated that a stable and constant expression of NGN2 in the microfluidic set-up does not allow for the formation of PAX6 positive cells. Next, we tried to dissect which are the main differences between mmRNA- and lentiviral-based approach that can account for the generation of this NSC population only with mmRNA delivery. The lentiviral system has the puro selection cassette, so the efficiency of neuronal conversion is very high, reaching a percentage of neuronal cells higher that 80%. We removed the selection step, but we did not observe any substantial difference in the differentiation outcome indicating that is not a matter of efficiency. When we partially activated NGN2 giving a short-term dox treatment, to mimic a pulse of NGN2 TF that can originate from an mmRNA transfection, we reduced neuronal conversion efficiency but we did not obtain any NSC, suggesting that partial activation of NGN2 is not responsible for the appearance of NSCs in culture.

Therefore, we focused our attention on the transient nature of mRNA. When NGN2 TF is delivered as mmRNA, the protein level may be fluctuating because of the intrinsic unstable nature of mRNA coupled with the need of daily transfections, as proved by the temporal profile of the percentage of NGN2 positive cells upon one or two daily transfections. It is known that in order to assure the maintenance of a NSC status, NGN2 should have an oscillatory pattern which is regulated by negative feedback from the oscillation of Hes1 (Kageyama et al., 2008; Shimojo et al., 2011). Meanwhile, when NGN2 expression level is constant, it leads to the activation of NeuroD and terminal differentiation into neurons (Borghese et al., 2010). Thus, NGN2 controls cell-autonomously neuronal differentiation inducing the expression and activity of NeuroD (Borghese et al., 2010), whereas controls progenitor maintenance non-cell-autonomously inducing Notch ligand expression and Notch pathway activation in adjacent cells. This leads to Hes1 expression in these adjacent cells, and consequent *NGN2* inhibition, maintaining their progenitor status via lateral inhibition leading to a spatial negative feedback (Chitnis, 1995).

Given the stochastic distribution of NGN2 mmRNA in the cells and the very short lifespan of mmRNA we can speculate that the delivery of NGN2 via mmRNA triggered an NGN2 oscillatory-like behavior similar to what can be observed in NSCs during development, giving rise to a NSC population *in vitro*. On the other hand, when NGN2 is delivered via an integrating system, the high and constant NGN2 protein level can mimic a neuronal differentiation program, leading to terminal differentiation into neurons. Future work is required and will be essential to demonstrate this hypothesis with target experiments aimed at dissecting the oscillatory nature of NGN2 in cells transfected in the microfluidics system and the consequent impact of oscillation on neural trajectory behavior.

In conclusion, we found that mmRNA-based NGN2 transcriptional programming is able to induce neuronal differentiation and to stabilize a distinct NSC population that with an integrating delivery system is not present or only transiently and briefly present.

Our results open a novel point of view for evaluating mRNA-based transcriptional programming strategies, considering that the administration of the same transcription factor either via mRNA or viral vector can lead to the generation of different cell populations. In addition, we envision that this mmRNA-based system can be used to study the regulation of cell fate using multiple TF, which can be finely tuned by a time specific delivery. In this way it will be possible to dissect the specific contribution of each TF in a desired transcriptional programming trajectory.

## Materials and Methods

### Cell Culture

The hiPSC line used has been generated and fully characterized in Luni et al. (2016). hiPSCs were maintained as feeder-free cells in TeSR-E8 (TeSR-E8 Basal Medium – STEMCELL Technologies, #05941) medium in 6-well plates coated with 0.5% of Matrigel (Corning Matrigel Matrix – Growth Factor Reduced, SACCO – Corning, #354230). Upon reaching 70% confluence, cells were dissociated in 0.5 nM EDTA and replated at 1:6–1:10 ratio. For neural cells differentiation, hiPSCs were dissociated at single cell level with TrypLE enzyme (TrypLE Select Enzyme (1X), no phenol red – Thermo Fisher Scientific, # 12563011) and seeded in the microfluidic chambers coated with 1% Matrigel.

### Microfluidic Platform

Microfluidic platforms were fabricated according to standard soft-lithographic techniques and molded in polydimethylsiloxane (PDMS) as previously described (Giulitti et al., 2013; Luni et al., 2016; Gagliano et al., 2019). Briefly, to obtain a single PDMS mold with five independent channels, Sylgard 184 (Dow Corning) was cured on a 200-μm-thick patterned SU-2100 photoresist (MicroChem). Each channel has a height of 200 μm and a surface suitable for cell culture of 27 mm^2^. The PDMS mold was punched at the edges of each channel with 1- and 3-mm punches to produce inlet and outlet holes, respectively, and sealed on a 75 × 25 mm microscope glass slide by plasma treatment. Channels were rinsed with isopropanol and distilled water to check proper flow before autoclaving.

### Virus Generation

All lentiviral vectors were handled in a class II biosafety laboratory. All plasmids were purchased on Addgene: pTet-O-Ngn2-puro (Addgene ID: 52047), M2-rtTA (Addgene ID: 20342), pRSV-REV (Addgene, #12253), pMDLg/pRRE (Addgene, #12251), and pMD2.G (Addgene, #12259). Lentiviral particles were generated with CaCl_2_ transfection. For transfection, 22 μg of pMD2.G, 15 μg of pRSV-Rev, 30 μg of pMDLg/pRRE and 75 μg of the lentiviral vector of interest (rtTA or Ngn2) were mixed in 2610 μl of TE buffer, then 290 μl of 2.5 M CaCl_2_ were added dropwise to the mixture and later 2900 μl of 2x HeBS buffer were added dropwise and incubated 5 min. Mixture was then added dropwise to two T175 flasks of HEK 293T cells (half of the mixture to each flask). Medium was changed 16 h after transfection and viruses were harvested 48 h after transfection, pelleted at 20,000 × *g* for 2 h at 4°C, resuspended in 100 μl DMEM overnight, aliquoted and kept at −80°C. Synapsin I-GFP lentivirus was kindly provided by Prof. Kokaia.

### Generation of Neural Cells *via* Lentiviral NGN2-Overexpression

On Day-2, hiPSCs were dissociated using TrypLE and plated, as single cells in TeSR-E8 medium supplemented with 10 μM Rock inhibitor (Y-27632 – CAS 146986-50-7 – Calbiochem, # 688000), in precoated microfluidic chambers. On Day-1 lentiviral transduction was performed at MOI of 0,045. To transduce hiPSCs, media was aspirated and replaced with TeSR-E8 containing the lentiviruses. On Day 0, dox (Sigma-Aldrich, #D9891) at 1 μg/ml concentration was added to N2B27 basal medium (DMEM/F-12, HEPES, Thermo Fisher Scientific, # 11330-032) supplemented with B-27 Supplement (1:50) (Thermo Fisher Scientific, # 17504-044) and N-2 Supplement (1:100) (Thermo Fisher Scientific, # 17502-001). On Day 1, media was replaced with fresh N2B27 basal medium containing 1 μg/ml puromycin (Puromycin Dihydrochloride – Thermo Fisher Scientific, # A1113803) and 1 μg/ml dox and changed daily. From Day 5, medium was daily replaced with N2B27 basal medium supplemented 10 ng/ml of Brain-derived neurotrophic factor (BDNF, PeproTech, #450-02), 10 ng/ml of Neurotrophin-3 (NT3, PeproTech, #450-03).

### Neural Cells Induction by *NGN2*-mmRNA

On Day-1, hiPSCs were treated with 0.2 ng/μl of B18R (Vaccinia Virus B18R Carrier-Free Recombinant Protein – eBioscience, # 34-8185-81) to reduce the immunological activity of the cells. On Day 0, hiPSCs were dissociated at single cell level using TrypLE and plated in TeSR-E8 medium supplemented with Ri and B18R. Cells were transfected right after dissociation and then daily with StemMACS NeuroG2 mRNA (Miltenyi Biotec, # 130-104-383) using StemMACS mRNA Transfection Kit (Miltenyi Biotec, # 130-104-463) according to protocol’s instruction described in Gagliano et al. (2019). Complexes of mmRNA and transfection reagent were added dropwise to channel inlet. Medium was replaced 6 h after transfection with TeSR-E8 containing B18R and Ri. On Day 1, medium was switched to N2B27 medium and changed daily till Day 4. From Day 5 till Day 10, medium was replaced with N2B27 medium supplemented 10 ng/ml of Brain-derived neurotrophic factor (BDNF), 10 ng/ml of Neurotrophin-3 (NT3).

### Immunofluorescent Protocol

Cells were fixed using 4% paraformaldehyde solution incubated for 10 min at room temperature. Blocking solution [PBS supplemented with 0.1% triton-X-100 (Sigma-Aldrich, #93426) and 5% donkey serum (Sigma-Aldrich, # S30-100ML)] was then added to each channel and left in incubation for 1 h at room temperature. Cells were incubated with primary antibodies diluted in blocking solution overnight at 4°C. Cells were then washed three times with TPBS (PBS with 0.1% triton-X-100), incubated for 1 h at room temperature with secondary antibodies diluted in blocking solution, washed again three times in PBST and mounted with mounting media. The following antibodies were used for our analysis: rabbit anti-NGN2 (D2R3D) (1:100, 13144S Cell Signaling), rabbit anti-PAX6 (1:300, 901301 BioLegend), mouse anti-OCT4-3/4 (C-10) (1:200, sc-5279 Santa Cruz Biotechnology) and mouse anti-TUBULIN β 3 (βIIITUBB) (1:5000, 801202 BioLegend). Appropriate Alexa Fluor 488 – and Alexa Fluor 594 -conjugated secondary antibodies (1:200, Jackson ImmunoResearch) were used. Nuclei were counterstained with Hoechst (1:5000 – Life Technologies, #H3570).

Images were taken by epifluorescence Leica DMI6000B microscope equipped with a mercury short-arc reflector lamp or Leica SP5 confocal microscope.

### Image Quantification

At least 10 random fields in 2 channels of 3 microfluidic devices in three independent experiments have been acquired and used for the analysis. Positive cells have been quantified starting from images acquired at the fluorescence microscope processed with the Image Processing Toolbox of MATLAB software (R2015b). All images were acquired using the same settings between experiments, pre-processed to increase the contrast and turned into black and white. The pre-processing function used has been the function *adapthisteq*, which performs contrast-limited adaptive histogram equalization avoiding the noise amplification. To turn the image into black and white we used the function *im2bw* where the threshold level was determined by the function *graythresh* applied to the image after contrast enhancement Noise and dirt elements of the images were removed by using the function *bwareaopen*. The resulting image was used as input of the function *bwlabel* which returns the number of distinct elements (representing in our case the number of nuclei) present in the processed image. An example of the different image analysis steps is reported in Supplementary Figure 3. IIITUBULIN-positive cells were manually determined counting the positive soma after merge with nuclei maintaining two different pseudocolors.

### Statistical Analysis

One-way ANOVA with Tukey’s *post hoc* correction was used for multiple comparisons, whereas student-t was used in case of comparisons between two groups. Data were analyzed using GraphPad software. SEM was used. *P*-values: ^∗^*p* < 0.05; ^∗∗^*p* < 0.01; ^∗∗∗^*p* < 0.005; ^****^p < 0.0001.

## Data Availability Statement

The raw data supporting the conclusions of this article will be made available by the authors, without undue reservation.

## Author Contributions

AT, CL, and NE designed the experiments and wrote the manuscript. AT and CL performed the experiments and analyzed the data. EG contributed to imaging analysis. IC produced the lentiviral vectors. OG and FM helped in microfluidic development and mRNA transfection optimization. MM contributed to design the experiments. ZK provided lentiviral vectors and contributed in the revision of the manuscript. NE supervised the project. All authors contributed to the article and approved the submitted version.

## Conflict of Interest

The authors declare that the research was conducted in the absence of any commercial or financial relationships that could be construed as a potential conflict of interest.
