# Supplementary material for: NGN2 mmRNA-Based Transcriptional Programming in Microfluidic Guides hiPSCs Toward Neural Fate With Multiple Identities
Source: Front Cell Neurosci. 2021 Feb 12;15:602888. doi: 10.3389/fncel.2021.602888 (PMC7928329; doi:10.3389/fncel.2021.602888)
Supplement: Supplementary file 1 [file Table_1.DOCX]

***Supplementary Material***

***
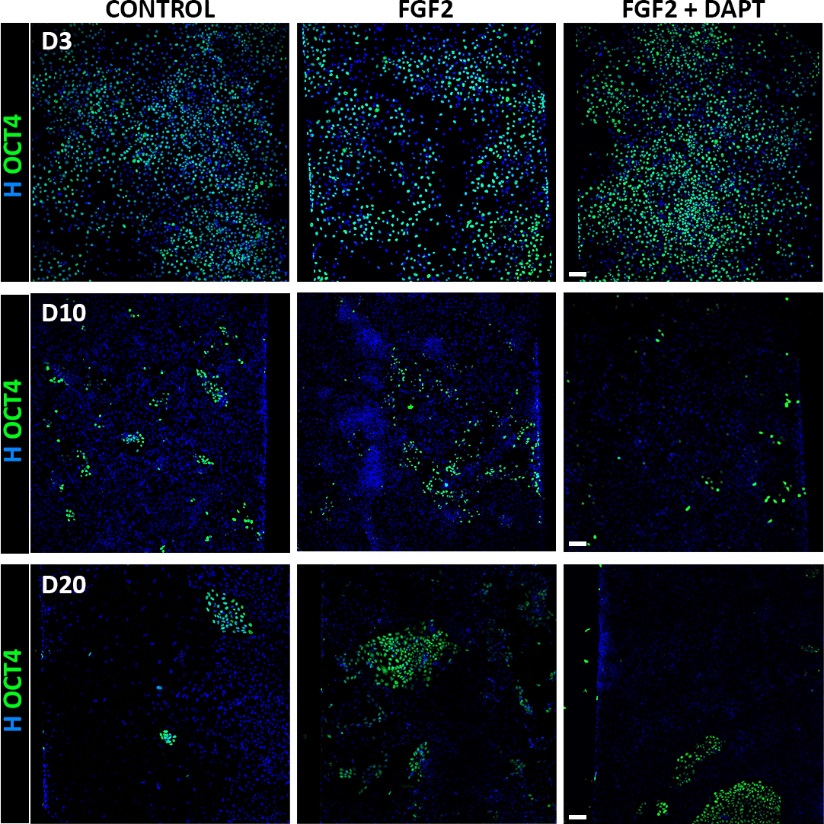
***

**Figure S1.** The number of pluripotent stem cells drops during time. Representative images of OCT4 staining (green) at Day 3, Day 10 and Day 20 in the presence or absence of FGF2 and DAPT. (scale bar=100 µm).


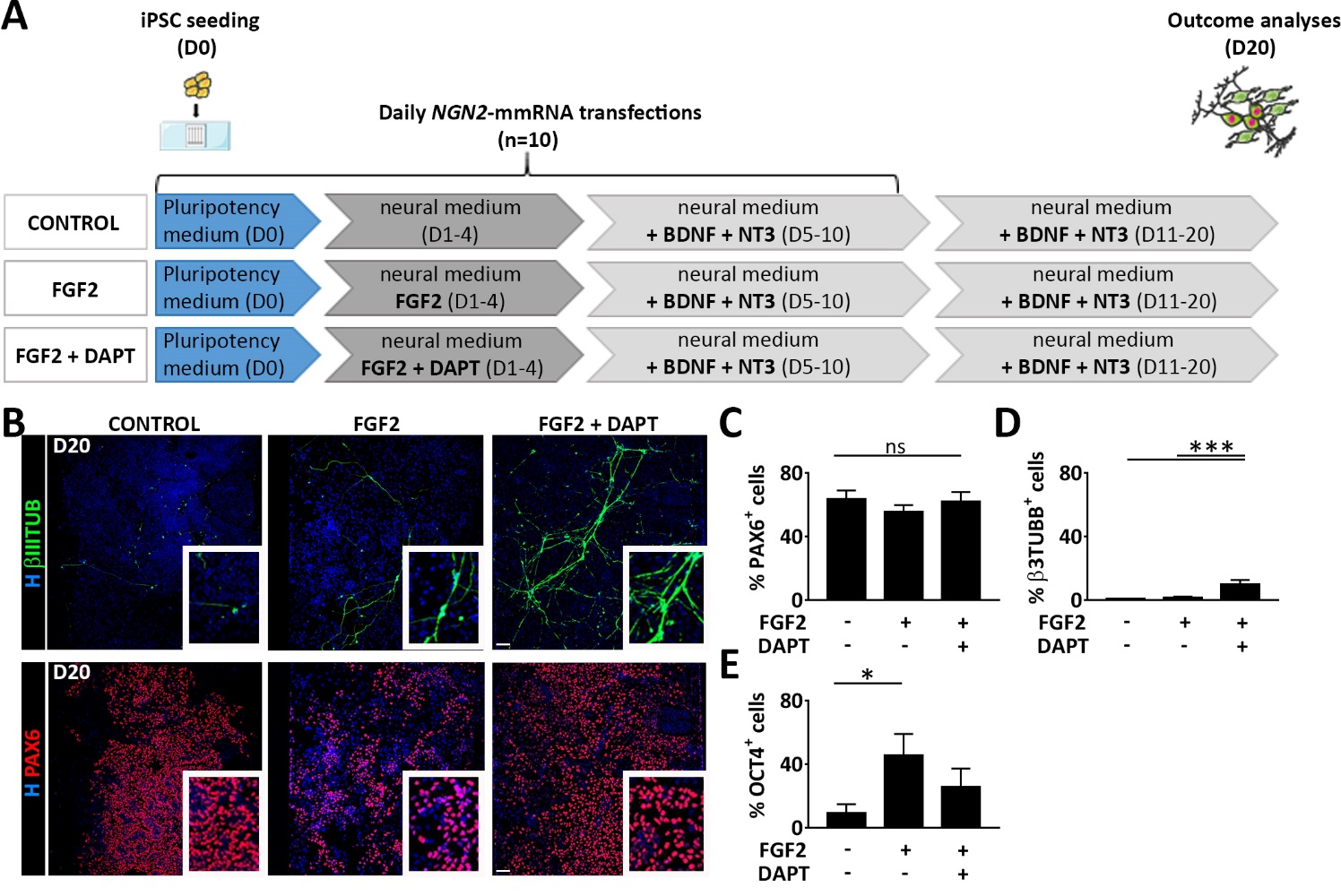


**Figure S2.** Treatment with FGF2 and DAPT improves neural conversion efficiency. **(A)** Experimental design. Note that FGF2 was used only from Day 1 to Day 4 and DAPT from Day 1 till the end of the differentiation protocol (Day 20). *NGN2*-mmRNA was administrated to iPSCs only from Day 0 to Day 10 **(B)** Representative images of βIIITUBULIN (top) and PAX6 (bottom) staining at Day 20 in the presence or absence of FGF2 and DAPT. Nuclei are counterstained with Hoechst (blue) (scale bar=100 μm). **(C-E)** Percentage of positive cells for PAX6 (C), βIIITUBULIN (D) and OCT4 (E) markers over the total cells at Day 10 in all the conditions. n=3 independent replicates [mean±SEM]


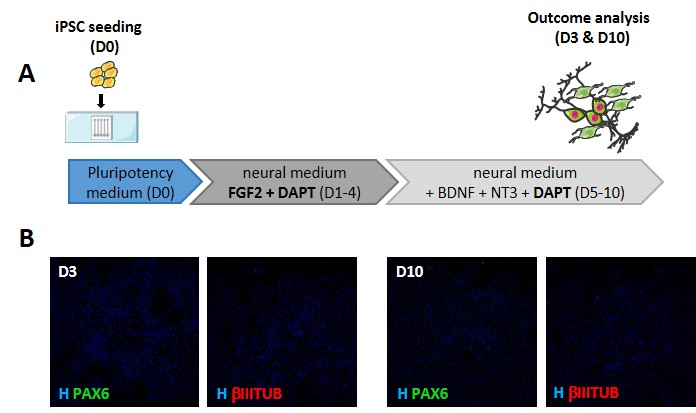


**Figure S3.** The medium per se in the absence of NGN2-mmRNA transfection doesn’t induce neural cells generation. **(A)** Experimental design. **(B)** Representative images of PAX6 (green) and βIIITUBULIN (red) staining at Day 3 and Day 10 in iPSCs treated only with neural media in the absence of NGN2 mmRNA transfection. (scale bar=100 µm).


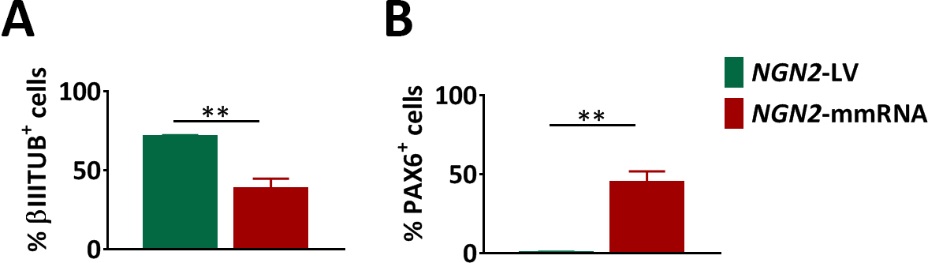


**Figure S4.** The *NGN2*-mmRNA transfections induce neural cells generation if compare with NGN2-LV transduction. Percentage of positive cells for βIIITUBULIN **(A)** and PAX6 **(B)** markers over the total cells at Day 10 in FGF2+DAPT condition after *NGN2*-LV and *NGN2*-mmRNA treatment, respectively. n=3 independent replicates [mean±SEM]


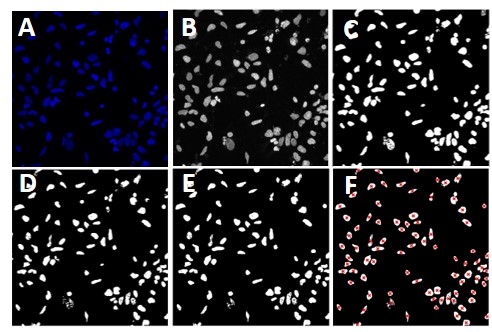


**Figure S5.** Steps of image analysis to automatically count the number of cells. **(A)** original image. **(B)** image after conversion to gray scale and filtering to enhance image contrast. **(C)** image after black and white conversion. **(D)** image after edge erosion. **(E)** image after very small size elements removal. **(F)** final image with counted nuclei highlighted by the red crosses.
